# Low-Pass Nanopore Sequencing of Plasma cfDNA Reveals Fragmentomic, Epigenomic, and Age-Associated Signatures Under Ultra-Low-Coverage Conditions

**DOI:** 10.3390/ijms27135739

**Published:** 2026-06-25

**Authors:** Andrey Eremin, Alexander Sergeev, Tsimur Hasanau, Maria Zvereva

**Affiliations:** 1 Department of Chemistry, Lomonosov Moscow State University, Moscow 119234, Russia; 2 Department of Bioengenering and Bioinformatics, Lomonosov Moscow State University, Moscow 119234, Russia; 3 Institute of Biomedical Chemistry, IBMC, Moscow 119121, Russia

**Keywords:** cfDNA, low-pass, nanopore sequencing, aging, fragmentomics, epigenomics

## Abstract

Circulating cell-free DNA (cfDNA) enables minimally invasive assessment of chromatin organization and DNA modifications. Whether such information can be reliably recovered under conditions of limited plasma input (below 1 mL) and ultra-low sequencing depth remains unclear. We performed low-pass whole-genome Oxford Nanopore sequencing (down to 0.01× coverage) of plasma cfDNA from young and elderly donors and jointly analyzed fragment length distributions and base modifications (5mC, 5hmC, 6mA). In parallel, we analyzed an enzymatically fragmented model DNA system to assess whether controlled in vitro fragmentation can reproduce cfDNA-like nucleosomal profiles and associated modification patterns. Despite shallow coverage, cfDNA samples displayed reproducible mono-, di-, tri-, and tetra-nucleosomal peaks, indicating that major fragmentomic features can be retained under ultra-low-coverage conditions. Modification-aware basecalling enabled exploratory quantification of global modification fractions across nucleosomal size classes and nomination of candidate group-specific modification loci. Overall, these results support the feasibility of low-pass nanopore sequencing as an exploratory framework for simultaneous cfDNA fragmentomic and epigenomic profiling in low-input studies.

## 1. Introduction

Circulating cell-free DNA (cfDNA) in blood plasma represents a minimally invasive source of genomic and epigenomic information and has emerged as a powerful substrate for biomarker discovery. In addition to genetic variation, cfDNA retains information about chromatin organization, nucleosome positioning, fragment-length distribution, and DNA modifications, enabling integrated analysis of fragmentomic and epigenomic features. Characteristic nucleosomal fragmentation patterns, including mono- and oligo-nucleosomal peaks, reflect the in vivo chromatin landscape, whereas cytosine and adenine modifications provide information related to gene regulation and cellular origin. As summarized in our recent review [1], cfDNA fragmentome and epigenome profiling provide a promising framework for developing cfDNA-based aging clock models and for evaluating age-associated physiological states. In particular, age-related alterations have been described both at the level of DNA methylation and nucleosome organization, supporting the concept that cfDNA captures systemic chromatin remodeling associated with aging.

Recent advances have substantially expanded the analytical scope of plasma cfDNA beyond mutation detection. Genome-wide cfDNA fragmentation patterns have been shown to reflect nucleosome positioning, transcription factor footprints, chromatin accessibility, and tissue or cell type of origin [2,3,4]. Low-coverage whole-genome sequencing approaches, including DELFI (DNA evaluation of fragments for early interception), have demonstrated that genome-wide cfDNA fragmentation profiles can provide diagnostic information even without deep sequencing of individual loci [5]. In parallel, cfDNA methylation profiling has emerged as a powerful strategy for tissue of origin inference and disease detection, supported by reference methylation atlases and methylation-based deconvolution methods [6,7,8]. Targeted methylation assays have also shown clinical potential for multi-cancer early detection and cancer signal localization [9,10].

Sequencing of cfDNA has become an established or rapidly developing tool in multiple clinical domains. In oncology, analysis of circulating tumor DNA (ctDNA) enables non-invasive detection of somatic mutations, monitoring of minimal residual disease, assessment of treatment response, and identification of emerging resistance mechanisms [11,12]. Fragmentomic and methylation-based approaches further improve tumor detection sensitivity and allow inference of tissue of origin [13]. In transplantation medicine, donor-derived cfDNA is widely used as a biomarker of graft injury and rejection, providing an early and quantitative indicator of organ damage without the need for invasive biopsy [14]. In prenatal diagnostics, analysis of fetal cfDNA in maternal plasma has transformed screening for chromosomal aneuploidies and monogenic disorders [15]. Additionally, cfDNA profiling is being explored in autoimmune [16], cardiovascular [17], and neurodegenerative diseases [18], where tissue-specific methylation patterns and fragmentation signatures can reflect cell-type-specific injury and systemic pathological processes.

Traditional approaches for methylation profiling of cfDNA rely on bisulfite conversion and short-read sequencing, which are associated with DNA degradation, loss of material, and limited preservation of native fragment-length information [19,20]. In contrast, single-molecule long-read sequencing technologies, including nanopore sequencing, enable direct detection of modified bases without bisulfite treatment while preserving native fragment-length information [21]. This allows fragmentomic and epigenomic features to be analyzed within the same individual cfDNA molecules. Nanopore sequencing of plasma cfDNA has been demonstrated to capture methylation and fragmentation patterns that correlate with organ-specific signals [22], and methylation classification remains feasible at genome coverage levels around 0.2× [23]. Recent nanopore-based cfDNA studies have further demonstrated single-molecule methylation profiling and methylation-based classification of cancer-derived cfDNA, highlighting the potential of long-read platforms for integrated cfDNA epigenomic analysis [24,25]. However, most such approaches rely on substantially higher sequencing depth than the ultra-low-coverage setting tested here.

Despite the rapid development of cfDNA-based aging clocks and nanopore-based cfDNA profiling, an important practical question remains insufficiently addressed: whether low-pass nanopore sequencing can retain informative fragmentomic and epigenomic features from low-input plasma cfDNA. In many experimental and clinical settings, especially pilot or exploratory studies, only limited plasma volumes are available, resulting in shallow whole-genome coverage. It is therefore essential to determine whether such data are sufficient to resolve major nucleosomal peaks and to nominate candidate age-associated modification loci.

The aim of the present study was to evaluate whether ultra-low-coverage nanopore sequencing of plasma cfDNA can recover major fragmentomic features and provide preliminary epigenomic signals in an exploratory comparison of young and elderly donors under low-input conditions. We evaluated whether shallow nanopore sequencing enables detection of nucleosomal fragmentation patterns and exploratory nomination of candidate differential base-modification loci. In addition, we employed a model DNA system to assess the extent to which controlled enzymatic fragmentation can reproduce cfDNA-like nucleosomal profiles and modification patterns. Together, these approaches provide insight into the feasibility of cfDNA-based epigenomic analysis for aging research when only limited amounts of plasma are available.

## 2. Results

Basecalled data filtration yielded comparable read counts (Table 1) for most libraries (approximately 0.46–0.64 million reads), with one sample showing reduced output (54,923 reads). The proportion of high-quality bases (Q30) ranged from 84.8% to 87.6% across the majority of cfDNA samples, indicating overall good basecalling performance; one sample (old_male_1158_batch1) exhibited a lower Q30 value (78.0%). Genome coverage relative to T2T-CHM13v2.0 was low (0.01–0.04×), consistent with the intended low-pass sequencing design.

Control samples based on the Raji cell line DNA produced substantially higher read counts (2.1–18.2 million reads) reflecting the greater amount of input DNA available for library preparation, with consistently high Q30 values (approximately 90.4–90.8%) and increased genome coverage (0.16–1.33×). Overall, the sequencing data demonstrate high basecalling quality and are suitable for downstream fragmentomic and DNA modification analyses under low-coverage conditions.

**Figure 1:**
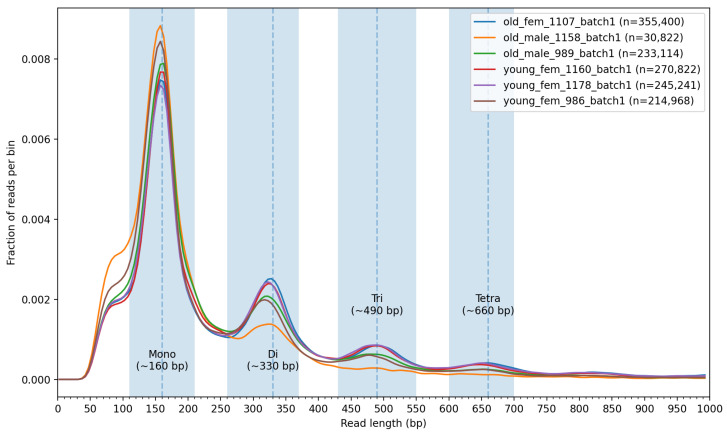
Read length distribution of cfDNA reads across six samples. The y-axis shows the fraction of reads per length bin. Canonical cfDNA nucleosomal peaks are visible at approximately 160 bp (mono), 330 bp (di), 490 bp (tri), and 660 bp (tetra), as indicated by vertical lines. The bins for separate analysis are shown as blue vertical lines.

Read length distributions of native cfDNA samples display a characteristic nucleosomal pattern with a dominant mononucleosomal peak centered at approximately 160 bp and clearly resolved di-, tri-, and tetra-nucleosomal peaks at around 330 bp, 490 bp, and 660 bp, respectively (Figure 1). The relative heights and positions of these peaks are consistent across individuals, indicating reproducible fragmentation profiles and preserved higher-order chromatin organization in circulating DNA.

Across nucleosomal size classes, the mean fraction of 5mC showed a gradual increase from mono- to tetra-nucleosomal fragments (3.497% to 4.115%), indicating a modest enrichment of cytosine methylation in longer cfDNA fragments. In contrast, 5hmC levels remained low across all groups and exhibited only minor variation (0.166–0.185%), without a clear monotonic trend. The fraction of 6mA ranged from 0.899% to 1.027%, with slightly elevated values in di- and tetra-nucleosomal fragments compared to mono- and tri-nucleosomal groups. Statistical significance of pairwise differences between fragment classes was assessed using two-sided Mann–Whitney U tests with Benjamini–Hochberg FDR correction (Table 2).

Within the region 120–220 nt (Table 3) methylated Raji DNA samples exhibited consistently higher mean 5mC fractions (4.186–4.740%) than non-methylated controls (3.229–3.573%) consistent with the expected effect of M.SssI treatment. Mean 5hmC levels remained low across all control samples (0.088–0.114%) and showed only minor variation between methylated and non-methylated DNA, indicating no substantial hydroxymethylation differences associated with enzymatic CpG methylation. Mean 6mA fractions showed greater variability across samples, including a pronounced difference between batch4 (0.845–0.898%) and batch6 (0.087–0.102%), suggesting a batch-dependent effect for 6mA measurements. In contrast, 5mC measurements were consistent across independently processed control samples, with low technical variability relative to the observed difference between methylated and non-methylated DNA (Table A1). Overall, these data confirm the expected increase in 5mC signal in enzymatically methylated controls while indicating substantially greater variability for 6mA than for cytosine modifications.

The pronounced inter-batch difference observed for 6mA indicates that this modification type is substantially more sensitive to technical variation than 5mC and 5hmC in the present nanopore workflow. Therefore, 6mA fractions and 6mA-based candidate loci were not interpreted as quantitatively robust biological differences. Instead, 6mA results were retained as exploratory observations illustrating the current limitations of simultaneous multi-modification calling under low-coverage conditions.

**Figure 2:**
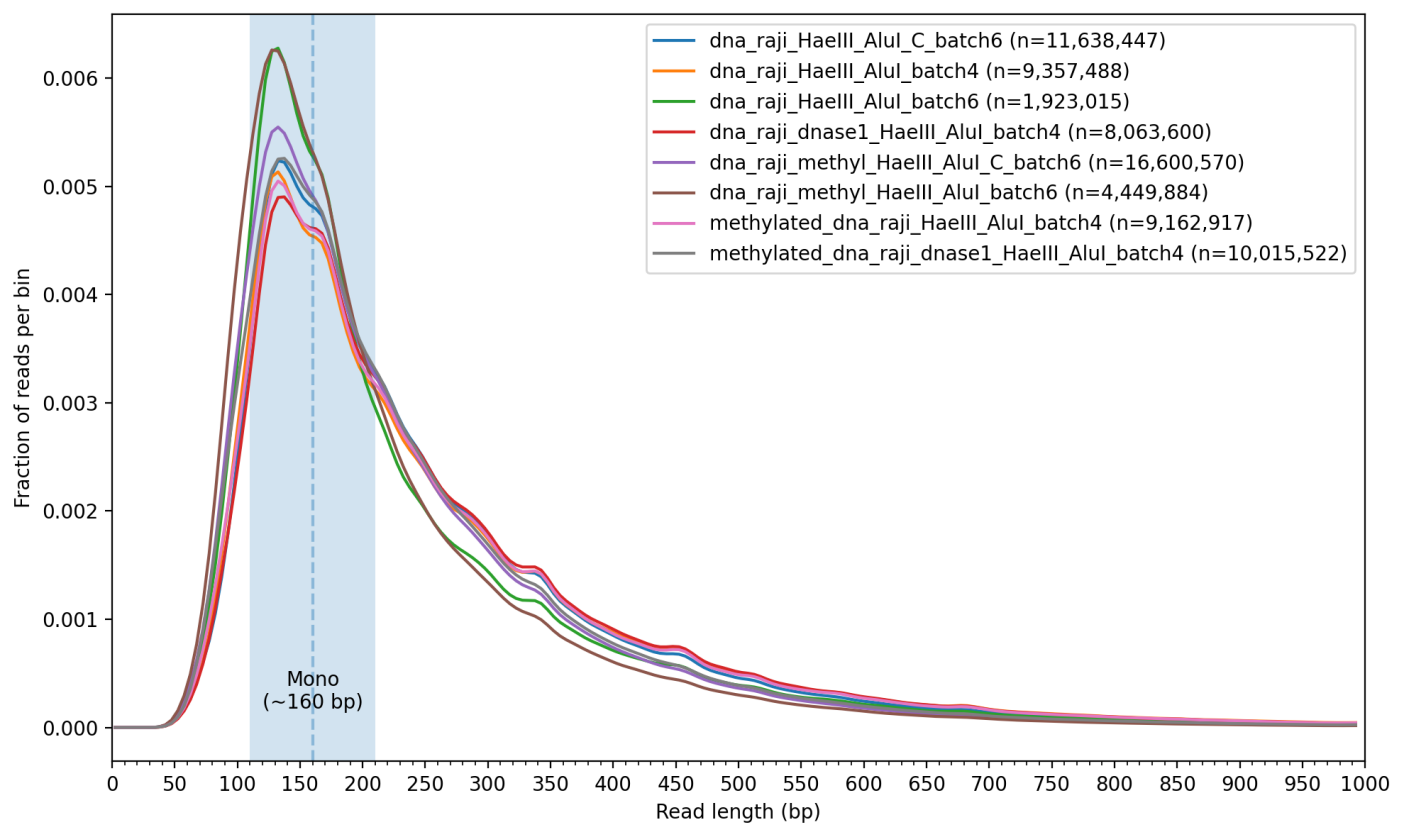
Read length distribution in model samples. The y-axis shows the fraction of reads per length bin.

Raji-derived model samples fragmented in vitro show a prominent peak within the mononucleosomal range (120–220 bp), confirming generation of cfDNA-like mononucleosomal fragments (Figure 2). However, this peak is shifted toward shorter fragment lengths compared to native cfDNA, with the maximum located closer to 140–150 bp. Furthermore, higher-order nucleosomal peaks are absent in the model samples, and the fragment length distribution beyond the mononucleosomal region exhibits a gradual decay rather than distinct periodic maxima.

Bidirectional intersection analysis nominated candidate group-specific modification sites for all three modification types (Table 4). The largest number of candidate sites was obtained for 6mA (4507 young canonical ∩ old modified and 4232 old canonical ∩ young modified), followed by 5mC (2812 and 2776 sites, respectively), whereas 5hmC yielded fewer loci (541 and 573 sites). Given the pronounced technical variability observed for 6mA in the control samples, the 6mA candidate sites should be interpreted with particular caution. Similar numbers of reciprocal 6mA candidates may reflect balanced group-level call sets, but they do not by themselves establish biological age-associated 6mA remodeling.

We identified 307 CpG sites showing an inverse 5mC/5hmC pattern between age groups. These loci were classified as 5mC-modified in young and canonical in old samples, while showing the opposite pattern for 5hmC. This pattern is compatible with a possible shift from 5mC toward 5hmC at the same genomic positions. However, because of the small cohort size and ultra-low coverage, these sites should be considered exploratory candidates for future validation of active cytosine demethylation dynamics possibly associated with aging.

Overlap analysis against previously reported age-associated CpG markers identified four exact matches between differential 5mC sites detected in low-pass nanopore cfDNA data and published aging-associated microarray-derived loci (Table 5). Three overlapping loci (cg03293883, cg03502979, and cg01033001) were found in the young modified ∩ old canonical set, whereas one locus (cg11122518) was identified in the reciprocal young canonical ∩ old modified set. Thus, overlapping sites were detected in both directions of the bidirectional intersection framework. The recovered loci included three intronic CpG sites and one site associated with the *ADPRHL1* locus. Although the absolute overlap with the reference catalog was limited, the recovery of previously reported age-associated methylation markers provides proof-of-principle support that low-pass nanopore sequencing captures biologically meaningful differential 5mC signals despite sparse genome coverage. Given the very limited callable CpG space imposed by low-pass sequencing, this level of concordance is consistent with sparse but non-random recovery of known aging-associated methylation loci.

Application of the LINE-1 promoter overlap filtering approach identified 16,755 cfDNA reads overlapping promoter regions of evolutionarily young LINE-1 subfamilies. Read counts and average 5mC levels for each sample are summarized in Table 6.

Because sample old_male_1158_batch1 had substantially lower coverage (355 reads), it was excluded from group-level comparison. Among the remaining samples, the mean 5mC level in elderly individuals was approximately 0.078, compared to approximately 0.093 in young individuals.

We examined the genomic distribution of 6mA, 5mC, and 5hmC modification sites across exonic, intronic, and intergenic regions in samples from young and old individuals. The results are summarized in Table 7. Across all three modification types, the majority of sites were located within intronic regions, followed by intergenic regions, whereas exonic regions contained the smallest fraction of modification sites. This distribution pattern was generally consistent between young and old samples for all nucleosomal fragment classes. No substantial age-associated shifts in genomic localization were observed for 6mA, 5mC, or 5hmC sites. Among the analyzed modifications, 5hmC showed the highest proportion of intronic localization and the lowest proportion of intergenic localization compared to 6mA and 5mC. In contrast, 6mA demonstrated the highest proportion of intergenic localization and the lowest proportion of exonic localization across nucleosomal classes.

To determine whether the observed genomic distribution of modification sites reflects the underlying distribution of cfDNA fragments, aligned reads were annotated across exonic, intronic, and intergenic regions. Reads were additionally separated into mono-, di-, tri-, and tetranucleosomal fragment classes. The corresponding results are summarized in Table 8. The genomic distribution of cfDNA fragments was highly similar between young and old samples. In both groups, most fragments overlapped intronic regions, followed by intergenic regions, whereas exonic regions represented the smallest fraction. A nucleosomal length-dependent trend was observed for exon-overlapping fragments. The fraction of exon-associated reads increased progressively with fragment size, from 6.8% in mononucleosomal fragments to 12.7% in tetranucleosomal fragments in young samples, and from 6.6% to 12.0% in old samples. In contrast, intronic and intergenic proportions remained relatively stable across nucleosomal classes.

## 3. Discussion

The progressive increase in the mean fraction of 5mC from mono- to tetra-nucleosomal fragments suggests that longer cfDNA molecules are preferentially derived from more highly methylated genomic regions. Cytosine methylation in mammalian genomes is strongly associated with transcriptionally inactive, compact chromatin, particularly constitutive and facultative heterochromatin [26]. Such regions are characterized by dense CpG methylation, reduced accessibility to transcription factors, and tighter nucleosome packing. Fragments spanning multiple nucleosomes are therefore more likely to originate from these structurally compact domains, where methylated DNA is protected within stable higher-order chromatin configurations. In contrast, shorter mononucleosomal fragments may be relatively enriched for DNA released from more open, transcriptionally active euchromatin, where CpG methylation levels are typically lower and chromatin is more accessible to nucleases.

The in vitro fragmentation of Raji genomic DNA using DNaseI followed by AluI+HaeIII digestion generated a mononucleosomal peak that resembles the dominant mononucleosomal peak observed in native cfDNA samples. In both datasets, the majority of fragments cluster within the 120–220 bp interval, supporting the use of this enzymatic approach as a model for cfDNA-like fragment sizes. Moreover, the mean fraction of 5mC per read within the mononucleosomal range is comparable between Raji-derived fragments and native cfDNA (approximately 3.5% in cfDNA), indicating that global cytosine methylation levels are preserved at a physiologically relevant scale. However, closer inspection of the fragment length distributions reveals systematic differences between native and model samples. In native cfDNA, the mononucleosomal peak is centered around ∼160 bp and is accompanied by well-defined di-, tri-, and tetra-nucleosomal peaks, reflecting higher-order nucleosomal organization in vivo. In contrast, Raji-derived samples exhibit a mononucleosomal peak that is shifted toward shorter fragment lengths, with the maximum located closer to ∼120–130 bp. Additionally, higher-order periodic peaks are absent in the model samples. Instead of discrete secondary maxima, the distribution shows a smoother decay toward longer fragments.

When comparing modification fractions within the mononucleosomal group, the mean 5mC level in native cfDNA is 3.497%, which is very similar to the values observed in the non-methylated Raji model samples (3.439%). In contrast, the mean fractions of 5hmC and 6mA in native cfDNA are 0.166% and 0.951%, respectively, whereas in the corresponding non-methylated Raji samples these values are lower, 0.09054% for 5hmC and 0.1022% for 6mA. Thus, while global 5mC levels in the model system closely approximate those of native cfDNA, other modification types differ in levels. Native cfDNA represents a composite pool of DNA fragments released from multiple tissues and cell types, predominantly hematopoietic cells but also endothelial and other peripheral sources [27]. Consequently, its modification profile reflects a weighted average of diverse epigenetic states across different lineages and differentiation stages. In contrast, the Raji model system is derived from a single transformed B-lymphoblastoid cell line [28] with a cancer-associated epigenetic landscape. Such cells are characterized by lineage-specific and tumor-associated alterations in DNA methylation and hydroxymethylation patterns, including global hypomethylation at repetitive elements and focal hypermethylation at regulatory regions. Therefore, even if global 5mC fractions appear similar, the underlying distribution and context of modifications are expected to differ between native cfDNA and the monoclonal Raji-derived DNA. This likely contributes to the observed discrepancies in 5hmC and 6mA levels.

Although restriction enzyme-based fragmentation of Raji DNA reproduces the mono-nucleosomal-like size class and approximates global 5mC levels, it does not replicate the nucleosomal positioning and higher-order chromatin structure characteristic of circulating cfDNA. The leftward shift of the mononucleosomal peak likely reflects differences between enzymatic cleavage patterns and endogenous nuclease activity in vivo, as well as the absence of chromatin-bound protection during fragmentation. Collectively, the model provides a reasonable approximation of cfDNA in terms of primary fragment size and overall methylation fraction, but it does not completely reproduce the fine-scale fragmentomic architecture observed in physiological samples.

The identified differential modification sites should be interpreted as preliminary candidate loci. The clinical cohort included only three young and three elderly individuals, which is insufficient for robust estimation of intra-group biological variance or for formal statistical inference at individual genomic loci. In addition, the ultra-low sequencing depth increases the probability of stochastic dropout of callable positions and makes the analysis sensitive to sample-specific coverage variation. Therefore, the intersection-based strategy used here was designed primarily as an exploratory approach to nominate group-specific modification candidates within the shared callable genomic space, rather than as a conventional differential methylation test. Within this limitation, the recovery of four exact overlaps with previously reported aging-associated CpG loci provides proof-of-principle support that ultra-low-coverage nanopore cfDNA sequencing can capture signals concordant with orthogonal methylation-array studies. However, this overlap should not be interpreted as independent validation of age-associated biomarkers. Instead, it indicates that the proposed workflow can recover biologically plausible candidate loci that require confirmation in larger, sex-balanced cohorts with increased sequencing depth and appropriate statistical modeling.

The observed co-localization of 5mC loss and 5hmC gain at the same aging-assosiated CpG sites is consistent with the canonical pathway of active DNA demethylation. In this process, 5mC is oxidized to 5hmC by TET enzymes, followed by further processing that can ultimately restore unmodified cytosine [29]. Importantly, the ability to detect such coordinated transitions is enabled by the use of nanopore sequencing, which allows direct identification of multiple DNA modifications within the same sequencing reads without chemical conversion or enrichment steps. This feature provides a unique opportunity to observe intermediate states of epigenetic processes, such as the conversion of 5mC to 5hmC and thus demethylation in the context of aging, at single-molecule resolution. This is particularly notable given the low sequencing coverage, which limits sensitivity and reduces the likelihood of detecting overlapping signals by chance.

Interestingly, one of the recovered overlapping loci mapped to *ADPRHL1* (cg03502979, located within the first intron of the gene), a gene not commonly represented among canonical epigenetic clock markers but functionally linked to biological processes relevant to aging. *ADPRHL1* has been implicated in regulation of focal adhesions, cytoskeletal organization, calcium handling, and ROCK–myosin II signaling, pathways associated with cellular senescence, tissue stiffness, and age-related functional decline [30]. The identification of an age-associated methylation locus within *ADPRHL1* may therefore be biologically meaningful, potentially reflecting epigenetic modulation of pathways involved in age-related mechanotransductive and structural remodeling. Although this observation does not establish a causal role for *ADPRHL1* in aging, it further supports that the loci recovered by the proposed intersection-based strategy are enriched for functionally relevant rather than purely stochastic signals.

**Figure 3:**
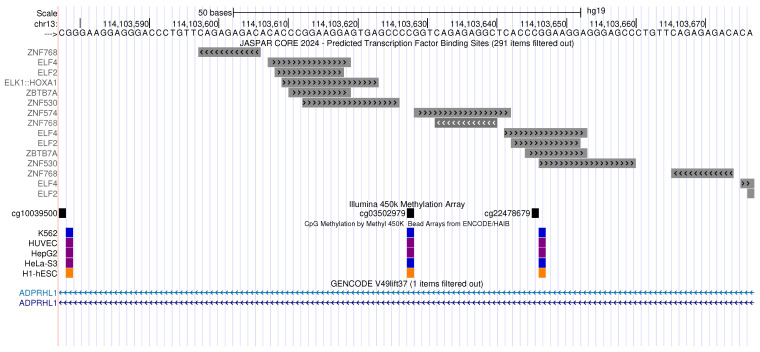
Genomic context of the age-associated CpG site cg03502979 within the first intron of *ADPRHL1*. UCSC Genome Browser view showing the position of cg03502979, predicted transcription factor binding sites from JASPAR CORE 2024 [31], and *ADPRHL1* gene annotation. The locus is located within a cluster of predicted transcription factor binding motifs, including ZNF574, ZNF530, ZBTB7A, ELF2, and ELF4, suggesting potential regulatory relevance of age-associated methylation changes at this site. CpG methylation tracks from ENCODE/HAIB [32] across multiple cell types (K562, HUVEC, HepG2, HeLa-S3, and H1-hESC) are shown for reference.

Notably, cg03502979 is positioned within a dense cluster of predicted transcription factor binding sites, including ZNF574, ZNF530, ZBTB7A, ELF2, and ELF4 (Figure 3), suggesting that this locus may reside within a regulatory hotspot rather than a functionally neutral intronic region. Because DNA methylation changes near transcription factor binding motifs can alter factor occupancy and local chromatin accessibility, age-associated methylation at this site could potentially influence transcription factor recruitment and thereby modulate *ADPRHL1* regulation. In particular, the proximity of cg03502979 to the predicted ZNF574 binding site raises the possibility that methylation changes at this locus may affect methylation-sensitive transcription factor interactions. Although this remains speculative, the positional relationship between this age-associated CpG and a cluster of putative regulatory motifs further supports the biological plausibility of this locus. For the three other CpGs, the predicted transcription factor binding sites are shown in Figure 3.

No clear evidence for a substantial age-associated difference in LINE-1 promoter methylation was observed in this analysis. Although the mean 5mC level was slightly higher in the young group compared to the elderly group (0.093 vs. 0.078), this difference was modest, and the direction of the trend should be interpreted cautiously. While the observed pattern is directionally consistent with the hypothesis that LINE-1 promoter methylation may decrease with age, these data do not support drawing firm conclusions regarding age-dependent methylation differences. A major limitation of this analysis is the very small number of biological samples (two elderly and three young individuals after exclusion of one low-coverage sample), which precludes meaningful statistical inference. With such limited replication, variability within groups cannot be robustly estimated, statistical power is extremely low, and formal significance testing would not be informative. In addition, variability among samples within groups was substantial and, in this dataset, appeared comparable to or greater than the observed difference between groups, further limiting interpretability. Another important limitation is that the observed difference in group means was small in magnitude (approximately 0.015 in absolute 5mC fraction), raising the possibility that it may reflect technical noise or biological variability rather than a reproducible age-associated effect. This concern is amplified by the low-pass sequencing design, which reduces sensitivity for detecting subtle epigenetic differences. Interpretation is further complicated by the nature of cfDNA itself, which represents a mixed signal originating from multiple tissues and cell types. As a result, any tissue-specific age-associated methylation differences at LINE-1 promoters may be diluted in the aggregate cfDNA signal. Together, these limitations indicate that the present analysis should be considered exploratory. Validation in larger cohorts, with increased sequencing depth and improved statistical power, will be required to determine whether age-related changes in LINE-1 promoter methylation can be robustly detected in cfDNA.

The analysis of genomic localization demonstrated that DNA modification sites are not randomly distributed across the genome, but instead are preferentially detected within intronic regions, whereas exonic regions contain the smallest proportion of modification sites. This pattern was consistently observed for 6mA, 5mC, and 5hmC across all nucleosomal fragment classes and remained generally stable between young and old samples. The absence of major age-associated shifts in genomic localization suggests that aging does not induce large-scale redistribution of DNA modifications across major genomic compartments. Instead, these findings support a model in which age-related epigenetic alterations occur predominantly at specific loci rather than through broad changes in genome-wide modification topology. Among the analyzed modifications, 5hmC exhibited the strongest intronic enrichment and the lowest intergenic representation. This observation is consistent with previous reports linking 5hmC to actively transcribed gene bodies and open chromatin regions [33]. In contrast, 6mA demonstrated the highest proportion of intergenic localization and the lowest exonic representation, suggesting that the genomic context of detected 6mA sites differs from that observed for cytosine modifications. Importantly, the genomic distribution of cfDNA fragments closely mirrored the distribution observed for modification sites. In both age groups, the majority of fragments overlapped intronic regions, followed by intergenic regions, whereas exonic fragments represented the smallest proportion. This similarity indicates that the apparent genomic localization of modification sites is strongly influenced by the underlying distribution of sequenced cfDNA fragments themselves. Consequently, a substantial fraction of the observed enrichment within specific genomic features may reflect fragmentation patterns, chromatin organization, mappability, and sequencing coverage rather than preferential biochemical targeting of modifications to those regions. The increase in exon-overlapping fragments with increasing nucleosomal fragment length suggests that longer cfDNA fragments may preferentially originate from genomic regions with distinct chromatin architecture or nucleosome organization. Exonic regions are often associated with higher GC content, increased nucleosome occupancy, and transcription-associated chromatin states, all of which may influence fragmentation behavior and mapping efficiency. Notably, this trend was observed in both young and old samples, indicating that it likely represents a general property of cfDNA fragmentation rather than an age-specific phenomenon. Overall, these findings emphasize that interpretation of modification landscapes in cfDNA requires careful consideration of the baseline genomic distribution of cfDNA fragments. The strong similarity between fragment and modification-site localization patterns suggests that global genomic enrichment analyses alone may not be sufficient to infer biological specificity of DNA modifications. Instead, locus-specific analyses and integration with additional epigenomic features will likely be necessary to identify biologically meaningful age-associated modification changes.

Technical variability is an important limitation of modification-aware nanopore sequencing in this study. The Raji control series revealed that 6mA measurements were strongly batch-dependent, with mean fractions decreasing from approximately 0.85–0.90% in batch4 to approximately 0.09–0.10% in batch6. This effect was much larger than the inter-batch variability observed for 5mC and 5hmC, indicating that 6mA calls are particularly sensitive to technical factors such as sample processing, library preparation, sequencing run, basecalling, or modification-calling behavior. Although all clinical cfDNA samples were processed using the same analytical workflow, this does not eliminate modification-specific technical noise. Therefore, 6mA results should be interpreted as sensitive to technical noise, and they require independent validation using larger cohorts, technical replicates, and orthogonal assays.

The bidirectional intersection analysis was designed to reduce the impact of sparse coverage and unequal callability by comparing modified and canonical calls within group-level callable sets. However, this approach does not constitute a formal statistical correction for batch effects or systematic modification-calling errors. In particular, if a modification type is prone to batch-dependent false-positive or false-negative calls, as suggested for 6mA by the Raji controls, such noise can influence the number and identity of candidate group-specific sites. For this reason, the candidate loci reported here should not be interpreted as validated differential modification sites. The 5mC candidates overlapping previously reported aging-associated CpG markers provide proof-of-principle support for the approach, whereas 6mA candidates are presented mainly as preliminary observations demonstrating the limitations of multidimensional modification calling under ultra-low-coverage conditions. The bidirectional intersection analysis has important false-positive and false-negative implications. False-positive candidate loci may arise from stochastic coverage variation, sample-specific modification calls, batch effects, or systematic modification-calling errors, particularly under ultra-low coverage. Conversely, false negatives may occur when true biological differences are not detected because the corresponding genomic positions are not covered or not confidently callable in one or more samples. Thus, the approach partially reduces callability imbalance by restricting comparisons to group-level callable sets, but it does not account for inter-individual variability or coverage uncertainty in the manner of a formal statistical model.

An important limitation is the composite cellular origin of plasma cfDNA. Under physiological conditions, circulating cfDNA is derived predominantly from hematopoietic lineages, but the relative contribution of different cell populations may change with age, inflammation, hematopoietic remodeling, and subclinical disease. Because DNA methylation and chromatin fragmentation patterns are highly cell-type-specific, apparent age-associated cfDNA modification signals may reflect either true epigenetic changes within a given cell type or changes in the relative abundance of cfDNA released from different cell types. Conversely, heterogeneous cell-type composition may dilute cell-intrinsic epigenetic aging signals. In the present pilot cohort, we did not have sufficient sample size, matched clinical metadata, blood cell counts, inflammatory markers, or sequencing depth to perform reliable cell type deconvolution. Therefore, the candidate age-associated loci reported here should be interpreted as composite plasma cfDNA signals rather than cell-intrinsic aging markers. This limitation is particularly important for the interpretation of cfDNA-based epigenetic clock signals, since such signals may integrate both molecular aging and age-associated changes in cfDNA tissue or cell-type composition. A major limitation of the present study is the small clinical cohort with a sex imbalance between age groups. The young group consisted only of female donors, whereas the elderly group included both female and male donors. Therefore, age and sex effects cannot be separated in the present dataset, and some of the observed group-specific modification patterns may reflect sex-related rather than age-related differences. This limitation is relevant for locus-specific DNA modification analysis, because DNA methylation patterns may differ by sex at autosomal as well as sex chromosome-linked regions. Validation in larger, sex-balanced cohorts will be required to distinguish true age-associated cfDNA modification signals from sex-related effects and other sources of inter-individual variability. Therefore, age, sex, inter-individual variability, and technical variability cannot be fully separated in the present dataset. This limitation is particularly important for locus-specific modification analysis and for LINE-1 promoter methylation analysis, where exclusion of one low-coverage elderly sample reduced the comparison to three young and two elderly individuals.

Ultra-low sequencing coverage is both a key feature and a major limitation of the present study. Coverage levels of 0.01–0.04× are sufficient to recover aggregate fragmentomic features, such as nucleosomal size distributions, because these signals are distributed across many cfDNA molecules. However, such coverage strongly limits locus-specific interpretation. Most genomic positions are not covered, many covered positions are represented by only one read, and the set of callable loci varies substantially between samples. As a result, modification detection at individual genomic positions is vulnerable to stochastic sampling, read-level uncertainty, and modification-calling noise. This increases the probability of false negatives, when true biological differences are missed because the relevant loci are not covered, and false positives, when candidate loci are driven by sparse or sample-specific calls.

The present findings should be interpreted in the context of recent developments in cfDNA fragmentomics and epigenomics described in the Introduction. Our work differs from these studies in its technical focus on ultra-low-coverage nanopore sequencing of native cfDNA. Rather than aiming to build a diagnostic classifier or a calibrated epigenetic clock, we evaluated whether several classes of cfDNA information could be recovered simultaneously from the same low-pass nanopore dataset. The preservation of nucleosomal fragment-size peaks supports the feasibility of fragmentomic profiling under these conditions, whereas the modification calls provide only preliminary epigenomic candidates because of sparse coverage, small cohort size, and modification-specific technical noise. This distinction is particularly important when comparing our results with recent cfDNA aging studies, which used larger cohorts and/or higher-resolution methylation profiling to identify cfDNA methylation aging signatures and cfDNA-based epigenetic clocks [34,35]. Our study should be regarded as a pilot feasibility analysis demonstrating what types of cfDNA fragmentomic and epigenomic signals can be recovered under ultra-low-coverage nanopore sequencing conditions.

## 4. Materials and Methods

### 4.1. Sample Annotations

Six blood plasma samples were obtained from the Orekhovich Institute of Biomedical Chemistry (Moscow, Russia) (Table 9). cfDNA was extracted from 1 mL of plasma using MagicPure^®^ Cell-Free DNA Kit II (TransGen Biotech Co., Ltd., Beijing, China) according to the manufacturer’s protocol. CfDNA concentration was monitored with Qubit 4 fluorometer (Thermo Fisher Scientific Inc., Waltham, MA, USA).

The protocol for collection of human blood samples and subsequent whole genome or whole exome sequencing was reviewed and approved by the Ethics Committee of the Russian Gerontology Clinical Research Center (protocol No. 59, 13 September 2022). All corresponding procedures were carried out in accordance with institutional guidelines and the Code of Ethics of the World Medical Association (Declaration of Helsinki).

Eight control DNA samples were generated from genomic DNA of the Raji cell line (Evrogen, Moscow, Russia) in two independent experimental batches (batch4 and batch6) to model different fragmentation and sample-processing conditions. For methylated controls, 1 µg of genomic DNA was treated with M.SssI DNA methyltransferase (SibEnzyme, Novosibirsk, Russia; 0.1 U/µL, 1 h, 37 °C) in a 50 µL reaction prior to fragmentation. Fragmentation was performed either by digestion with an AluI/HaeIII endonuclease mix alone (SE buffer G, SibEnzyme; 0.1 U/µL of each enzyme, 37 °C, 60 min) or by sequential treatment with DNase I (0.002 U/µL, 37 °C, 2 min) followed by AluI/HaeIII digestion. DNA was purified after each enzymatic step using GentaPure Cleanup magnetic beads (Genterra, Moscow, Russia).

Batch4 included four model samples designed to evaluate the effects of M.SssI methylation and DNase I pretreatment under independent library preparation and sequencing conditions. Batch6 included four additional independently processed samples that reproduced the same fragmentation framework while additionally modeling cfDNA extraction from surrogate plasma buffer (PBS + 4% BSA + 5 mM EDTA, DNase-free) using the MagicPure Cell-Free DNA Kit II (TransGen Biotech Co., Ltd., Beijing, China). Thus, the final control set included two independent experimental runs differing by M.SssI treatment, DNase I pretreatment, and cfDNA extraction conditions (Table 10).

### 4.2. Library Preparation

PromethION sequencing libraries were prepared from cfDNA and model samples using 6 ng cfDNA per sample according to the SQK-NBD114.24 Ligation Sequencing gDNA-Native barcoding kit 24 V14 PromethION protocol (Oxford Nanopore Technologies, Oxford, UK). The sequencing mix was prepared with 35 µL of the barcoded DNA library. The mix was added to PromethION R10.4.1 flow cell loaded into P2 Solo sequencing unit (Oxford Nanopore, Oxford, UK). The sequencing run lasted 24 h and yielded sequence data in POD5 format. MinKNOW software (version 5.7.2) was used to initialize and monitor the sequencing run.

### 4.3. Basecalling and Data Filtration

Raw nanopore sequencing data in POD5 format were basecalled using dorado basecaller v1.3.1 [36] with the dna_r10.4.1_e8.2_400bps_sup@v5.2.0/ basecalling model. In addition to canonical basecalling, modification-aware models were used to identify DNA base modifications, including N(4)-methylcytosine (4 mC, model dna_r10.4.1_e8.2_400bps_sup @v5.2.0_4mC_5mC@v1), 5-methylcytosine and 5-hydroxymethylcytosine (5mC and 5hmC, model dna_r10.4.1_e8.2_400bps_sup@v5.2.0_5mC_5hmC@v2), and N6-methyladenine (6mA, model dna_r10.4.1_e8.2_400bps_sup@v5.2.0_6mA@v1). For each sequencing batch, a single BAM file was produced containing aligned cfDNA reads with per-read DNA modification information encoded in standard BAM modification tags. The resulting modBAM file was filtered by Q-score (tag “qs” >20) and read length (50< read length <3000 bp) using samtools v1.23.1 [37]. Barcodes and technical library preparation sequences were removed using the dorado trim module with the corresponding sequencing kit definition SQK-NBD114-24. Dorado aligner was used to align trimmed reads against the human T2T-CHM13v2.0 reference genome [38]. Modkit v0.6.1 [39] pileup was used to obtain modBED files with parameter –filter-threshold 0.8 to get only high confidence calls.

### 4.4. Read-Level Fragmentome and Epigenome Analysis

Read-level modification statistics were computed from an aligned BAM file using python’s pysam [40]. Only primary alignments were retained. Reads flagged as unmapped, secondary, or supplementary were excluded. For each filtered read, the forward-oriented nucleotide sequence was retrieved. The total numbers of adenines (A) and cytosines (C) in the sequence were counted to serve as denominators for modification fraction calculations. Modified bases were obtained from the “modified_bases_forward” attribute. For each combination of canonical base, strand, and modification code, all reported modification sites were examined. Each site is associated with a probability value in the range [0, 255] (ML tag in modBAM file produced by dorado basecaller), which was rescaled to [0, 1] by division by 255. A modification call was accepted if the rescaled probability was greater than or equal to the predefined threshold $p_{\mathrm{mod}}=0.8$. The number of accepted sites was summed for each modification code across strands and base contexts.

Three modification types were quantified: 6mA, 5mC and 5hmC. For each read, modification fractions were calculated as

$${\mathrm{frac}}_{\text{6mA}}=\frac{n_{\text{6mA}}}{N_{A}},\;{\mathrm{frac}}_{\text{5mC}}=\frac{n_{\text{5mC}}}{N_{C}},\;{\mathrm{frac}}_{\text{5hmC}}=\frac{n_{\text{5hmC}}}{N_{C}},$$

where $n_{x}$ denotes the number of confidently detected modified bases of type *x*, $N_{A}$ is the total number of adenines in the read, and $N_{C}$ is the total number of cytosines.

Each read was checked for being aligned to an interspersed repeat sequence such as LINE, SINE, LTR and microsatellite. The annotation of repeats produced by RepeatMasker [41] and provided by the authors T2T-CHM13v2.0 of was used (file name chm13v2.0_RepeatMasker_4.1.2p1.2022Apr14.bed.gz). Tabix [42] was used to index the RepeatMasker’s annotation.

After construction of the read-level table, fragments were stratified according to their length to approximate nucleosomal organization. Size ranges were defined as follows: mononucleosomal fragments (110–210 bp), dinucleosomal fragments (260–370 bp), trinucleosomal fragments (430–550 bp), and tetranucleosomal fragments (600–700 bp). Reads falling within these intervals were assigned to the corresponding category, enabling downstream comparisons across nucleosomal fragment classes.

### 4.5. Identification of Age-Associated Differential Modification Sites

For each sample, canonical and modified base calls (6mA, 5mC, and 5hmC) were exported as BED files derived from modBAM alignments using Modkit pileup. Because the data was generated under low-pass sequencing conditions, modification calls were first merged across biological replicates within each age group (young and old) to generate group-level union sets of modified and canonical positions separately for each base type. This union-based strategy increased sensitivity and mitigated stochastic dropout of callable sites due to limited coverage. To control for coverage and callability differences between groups, intersections between young and old union sets were computed to define a shared genomic space for comparison. Candidate group-specific modification sites were identified by intersecting positions classified as modified in one age group with positions classified as canonical in the opposite age group. This bidirectional intersection strategy (young_modified ∩ old_canonical and young_canonical ∩ old_modified) was applied independently for each modification type. The purpose of this approach was to restrict comparisons to positions represented in both group-level call sets and to reduce the effect of stochastic dropout caused by ultra-low sequencing coverage. However, this strategy should be regarded as an exploratory filtering procedure rather than a formal differential modification test. It does not fully remove systematic modification-calling bias, batch effects, or modification-specific technical noise.

**Figure 4:**
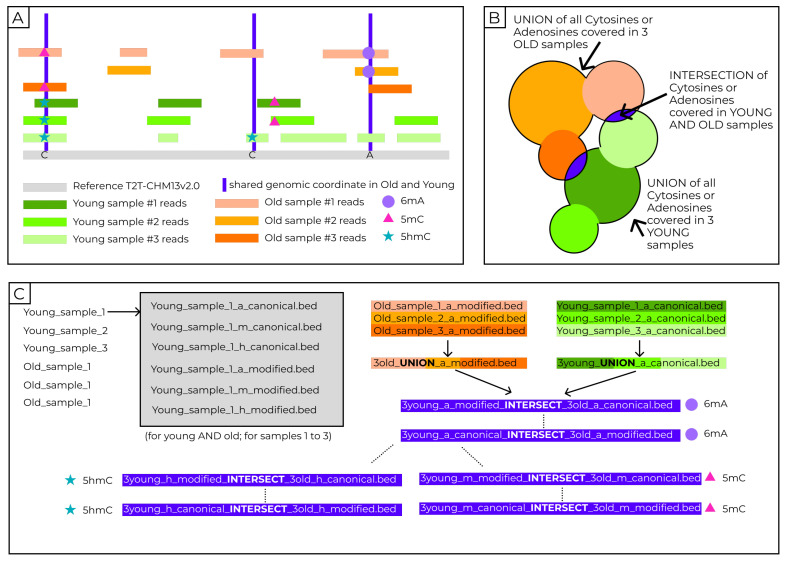
Workflow for comparative analysis of age-associated DNA modifications in young and old samples. (**A**) Schematic representation of canonical and modified cytosine/adenosine positions detected in individual young (green) and old (orange) samples relative to the reference genome (gray). Colored markers denote different modification types (6mA, 5mC, 5hmC). (**B**) Strategy for defining age-specific sites. For each age group, modification calls are first merged across biological replicates to generate group-level union sets. Intersections between young and old unions are then used to identify shared and age-specific modified or canonical positions. (**C**) File-level implementation of the pipeline. Individual sample BED files (canonical and modified) are merged to obtain group-specific union sets (3young_UNION and 3old_UNION). Pairwise intersections between modified and canonical sets across age groups are computed to derive regions consistently modified in one group but canonical in the other, enabling robust identification of age-associated differential modification sites.

To assess whether differentially methylated sites identified from low-pass nanopore cfDNA sequencing overlapped previously reported age-associated methylation markers, a reference set of 262,317 age-associated CpG sites (8540 reported by [43], 162 reported by [44], 987 reported by [45], 252,628 reported by [46]) was compiled and mapped from Illumina HumanMethylation450K annotations (GRCh37/hg19, columns CHR: Chromosome containing the CpG (Build 37); MAPINFO: Chromosomal coordinates of the CpG (Build 37)) to T2T-CHM13v2.0 coordinates using LiftOver [47]. These reference loci were compared against differentially methylated sites identified in the Young vs. Old comparison, represented by the sets young canonical ∩ old modified and old canonical ∩ young modified. Overlap analysis was first performed using exact coordinate matching (single-base overlap). Because previously reported age-associated loci are predominantly described for cytosine methylation, validation against published aging markers was performed for 5mC differential sites. Overlapping loci were annotated with their original probe identifiers and gene or feature context and were used as a proof-of-principle benchmark for the ability of low-pass nanopore cfDNA data to recover known age-associated methylation markers (Figure 4).

To assess whether age-associated changes in 5mC are linked to active DNA demethylation, we specifically analyzed overlap between CpG sites losing 5mC with age and sites gaining 5hmC. The latter is an intermediate product of TET-mediated oxidation of 5mC, catalyzed by TET enzymes. We selected CpG sites classified as modified in young and canonical in old samples for 5mC (putative loss of methylation), and intersected them with CpG sites classified as canonical in young and modified in old samples for 5hmC (putative gain of hydroxymethylation). This analysis was designed to identify loci consistent with a transition from 5mC to 5hmC, indicative of active demethylation.

### 4.6. LINE-1 Promoter Methylation Analysis

To test the hypothesis that LINE-1 promoters are more strongly methylated in young individuals than in elderly individuals, methylation levels in promoter regions of evolutionarily young LINE-1 elements were assessed using nanopore cfDNA sequencing data. Evolutionarily young LINE-1 subfamilies were selected because they retain relatively intact promoter regions and include potentially active elements subject to epigenetic repression.

RepeatMasker annotation was used to identify LINE-1 elements belonging to evolutionarily young LINE-1 elements (L1HS, L1PA2, L1PA3, L1PA4, L1PA5, and L1PA6). Only copies containing a substantially preserved promoter region were retained, defined as elements covering more than 50% of the promoter consensus corresponding to positions 0–900 of the LINE-1 consensus sequence. For each selected element, genomic coordinates of the corresponding promoter fragment and its strand orientation were determined, and promoter interval lists were constructed for each chromosome.

Aligned cfDNA reads were extracted from BAM files and processed individually. Unmapped, secondary, and supplementary alignments were excluded. For each remaining read, the nucleotide sequence was extracted, and the total numbers of adenines and cytosines were calculated. DNA modifications (6mA, 5mC, and 5hmC) were identified using modification probabilities of at least 0.8. Each read alignment was represented as a set of aligned blocks derived from its CIGAR structure, accounting for split alignment segments caused by insertions or other alignment discontinuities.

For each read and each LINE-1 promoter interval, the cumulative overlap between all alignment blocks and the promoter interval was calculated, together with the total aligned read length. The fraction of promoter overlap was computed as:

(1) $$\mathrm{Overlap}\;\mathrm{fraction}=\frac{\mathrm{cumulative}\;\mathrm{overlap}\;\mathrm{with}\;\mathrm{promoter}}{\mathrm{aligned}\;\mathrm{read}\;\mathrm{length}}$$

Only reads with overlap fraction ≥0.5 were retained. For each retained read, the following information was recorded: read identifier, sample barcode, LINE-1 subfamily, promoter coordinates and strand orientation, overlap fraction, read length, and per-read fractions of 6mA, 5mC, and 5hmC. Sample-level methylation estimates were calculated as the mean fraction of methylated cytosines among all cytosines in reads overlapping LINE-1 promoters.

### 4.7. Annotation of Modified Sites and cfDNA Fragments Across Genomic Features

To evaluate the genomic localization of detected DNA modifications, modified sites were annotated with respect to exonic, intronic, and intergenic regions. The analysis was performed using the CHM13v2.0 RefSeq Liftoff v5.2 genome annotation in GFF3 format. Exon and gene coordinates were extracted from the GFF3 file using awk scripts. Since GFF3 coordinates are 1-based and BED coordinates are 0-based, the start coordinate was converted by subtracting one. Exonic regions were extracted from records with feature type “exon”, and gene regions were extracted from records with feature type “gene”. Intronic regions were generated by subtracting “exon” coordinates from “gene” coordinates using bedtools subtract. Intergenic regions were generated as the complement of gene coordinates across the genome using bedtools complement with a genome size file derived from the reference FASTA index. All BED files were sorted using the Unix sort utility prior to downstream analysis. For each modification type, files containing all modified sites detected in “young” and “old” samples were used: young_6mA_modified.bed, old_6mA_modified.bed, young_5mC_modified.bed, old_5mC_modified.bed, young_5hmC_modified.bed, and old_5hmC_modified.bed. Each of these files represents the union of all sites classified as modified for the corresponding modification type and age group. Overlap between modified sites and genomic features was computed using “bedtools intersect” with the -u option. Separate intersections were performed for exons, introns, and intergenic regions. A given site was allowed to overlap multiple feature categories due to overlapping gene models and alternative transcript structures. For each modification type and age group, the number of sites overlapping exonic, intronic, and intergenic regions was counted. Percentages were calculated relative to the total number of modification sites within the corresponding modification type and age group.

To evaluate whether the genomic distribution of DNA modification sites reflects the underlying distribution of cfDNA fragments, aligned reads were annotated with respect to exonic, intronic, and intergenic regions. For group-level analyses, individual BAM files from young and old samples were merged using “samtools merge”. Only primary alignments were retained using “samtools view” by excluding unmapped, secondary, and supplementary alignments. The resulting BAM files were converted to BED format using “bedtools bamtobed”. Reads were then divided into nucleosomal fragment classes according to their aligned fragment length. Size ranges were defined as follows: mononucleosomal fragments, 110–210 bp; dinucleosomal fragments, 260–370 bp; trinucleosomal fragments, 430–550 bp; and tetranucleosomal fragments, 600–700 bp. Reads falling within these intervals were assigned to the corresponding category. The same genomic annotation files used for modification-site annotation were applied to fragment annotation. Overlaps between cfDNA fragments and exonic, intronic, and intergenic regions were calculated using “bedtools intersect” with the -u option. A fragment was allowed to overlap more than one feature category. For each age group and nucleosomal class, the number of fragments overlapping each genomic feature was counted. Percentages were calculated relative to the total number of reads within the corresponding nucleosomal fragment class.

To account for variability between biological replicates, the analysis was additionally performed independently for each individual sample. For each sample, proportions of exon-, intron-, and intergenic-overlapping reads or modification sites were calculated separately. Group-level summary statistics were then obtained by averaging proportions across samples within each age group. For each genomic category, mean percentages and corresponding 95% confidence intervals were calculated using the Student’s t-distribution. Confidence intervals were estimated as:

(2) $$\bar{x}\pm t\times \frac{s}{\sqrt{n}}$$

where $\bar{x}$ is the sample mean, *s* is the sample standard deviation, *n* is the number of biological replicates, and *t* is the critical value of the t-distribution. Since each age group included three biological replicates (n=3), the number of degrees of freedom was n−1=2. Therefore, for a two-sided 95% confidence interval, the critical value was $t_{0.975,2}=4.303$.

## 5. Conclusions

This pilot study supports the feasibility of extracting major fragmentomic and exploratory epigenomic signals from native plasma cfDNA using nanopore sequencing under ultra-low-coverage conditions down to 0.01× genome coverage. Despite sparse genome representation and a limited sample cohort, cfDNA reads retained characteristic nucleosomal fragmentation patterns, including mono-, di-, tri-, and tetra-nucleosomal peaks.

The analysis also nominated candidate group-specific DNA modification loci, including several 5mC sites overlapping previously reported aging-associated CpG markers. This concordance provides proof-of-principle evidence that low-pass nanopore cfDNA sequencing can recover methylation signals consistent with orthogonal array-based studies. However, due to the very small cohort size, limited statistical power, sex imbalance between age groups, and ultra-low coverage, these loci should be considered preliminary candidates rather than validated age-associated biomarkers. Similarly, loci showing apparent 5mC loss together with 5hmC gain should be interpreted as exploratory signals potentially consistent with cytosine demethylation dynamics, requiring validation in larger cohorts.

The model DNA system showed that controlled enzymatic fragmentation can reproduce cfDNA mono-nucleosomal-like fragments, but does not recapitulate the higher-order nucleosomal architecture of native plasma cfDNA.

Together, these findings support the feasibility of low-pass nanopore sequencing for cfDNA-based fragmentomic and epigenomic analysis, while further improvements in coverage, cohort size, signal calibration, and analytical methods will be required to increase sensitivity and validate age-associated markers.

## Figures and Tables

**Table 1:** Sequencing statistics and coverage relative to T2T-CHM13v2.0.

Sample Name	Total Reads	Q30 (%)	T2T-CHM13v2.0 Coverage, ×
young_fem_1178_batch1	481,714	87.59	0.03
old_male_989_batch1	643,282	85.06	0.03
old_fem_1107_batch1	604,477	87.01	0.04
young_fem_1160_batch1	54,923	87.13	0.03
young_fem_986_batch1	493,445	84.82	0.02
old_male_1158_batch1	464,874	78.01	0.01
dna_raji_HaeIII_AluI_batch6	2,160,296	90.42	0.16
dna_raji_methyl_HaeIII_AluI_batch6	4,953,520	90.43	0.34
dna_raji_HaeIII_AluI_C_batch6	12,586,636	90.78	1.01
dna_raji_methyl_HaeIII_AluI_C_batch6	18,162,037	90.53	1.33
dna_raji_HaeIII_AluI_batch4	10,184,339	90.44	0.85
dna_raji_dnase1_HaeIII_AluI_batch4	8,733,470	90.60	0.73
methylated_dna_raji_HaeIII_AluI_batch4	9,975,888	90.71	0.82
methylated_dna_raji_dnase1_HaeIII_AluI_batch4	11,029,979	90.08	0.83

**Table 2:** Mean fraction (%) of 5mC, 5hmC, 6mA in all reads of real cfDNA samples in Mono-, Di-, Tri- and Tetra-nucleosomal groups. Pairwise comparisons were performed using two-sided Mann–Whitney U tests. *p*-values were adjusted for multiple testing using the Benjamini–Hochberg false discovery rate (FDR) method.

	Mono	Di	Tri	Tetra
Mean fraction (%) of 5mC in all reads	3.497	3.961	4.060	4.115
Mean fraction (%) of 5hmC in all reads	0.166	0.181	0.173	0.185
Mean fraction (%) of 6mA in all reads	0.951	1.019	0.899	1.027

**Table 3:** Mean modification fractions (%) within the 120–220 nt region in control fragmented DNA samples.

Sample	Region (120–220 nt)		
	Mean_frac_5mC	Mean_frac_5hmC	Mean_frac_6mA
dna_raji_HaeIII_AluI_batch4	3573	0.10408	0.87261
dna_raji_dnase1_HaeIII_AluI_batch4	3483	0.10138	0.85284
methylated_dna_raji_HaeIII_AluI_batch4	4740	0.11377	0.84541
methylated_dna_raji_dnase1_HaeIII_AluI_batch4	4492	0.10580	0.89778
dna_raji_HaeIII_AluI_C_batch6	3229	0.08834	0.08651
dna_raji_HaeIII_AluI_batch6	3439	0.09054	0.10221
dna_raji_methyl_HaeIII_AluI_C_batch6	4246	0.09745	0.09376
dna_raji_methyl_HaeIII_AluI_batch6	4186	0.09737	0.09307

**Table 4:** Number of age-associated differential modification sites identified by bidirectional intersection between canonical and modified positions in young and old groups for 6mA, 5hmC, and 5mC. Additionally, overlap between age-associated 5mC and 5hmC sites is shown.

Comparison	Direction	Number of Sites
6mA	young canonical ∩ old modified	4507
6mA	old canonical ∩ young modified	4232
5hmC	young canonical ∩ old modified	541
5hmC	old canonical ∩ young modified	573
5mC	young canonical ∩ old modified	2812
5mC	old canonical ∩ young modified	2776
5mC vs. 5hmC	young m modified ∩ old h modified	307

**Table 5:** Overlap between differential 5mC sites identified in low-pass nanopore cfDNA data and previously reported age-associated CpG markers from array-based studies.

Direction	ProbeID	Gene/Feature	CHM13v2.0	hg19
young modified ∩ old canonical	cg03293883	Non-coding DNA	chr6:86632142–86632143	chr6:86124741–86124742
young modified ∩ old canonical	cg03502979	1st Intron of *ADPRHL1*	chr13:112708155–112708156	chr13:114103627–114103628
young modified ∩ old canonical	cg01033001	Non-coding DNA	chr14:88398671–88398672	chr14:94637733–94637734
young canonical ∩ old modified	cg11122518	Non-coding DNA	chr15:75167865–75167866	chr15:77599760–77599761

**Table 6:** Distribution of reads overlapping LINE-1 promoters and average 5mC methylation levels across samples.

Sample	Number of Reads	Mean 5mC Level
old_fem_1107_batch1	4538	0.1007
old_male_1158_batch1	355	0.1176
old_male_989_batch1	2874	0.0562
young_fem_1160_batch1	3445	0.0926
young_fem_1178_batch1	3000	0.1111
young_fem_986_batch1	2543	0.0750

**Table 7:** Genomic distribution of 6mA, 5mC, and 5hmC modification sites across exonic, intronic, and intergenic regions in cfDNA fragments of different nucleosomal classes. Values are presented as mean percentages across biological replicates with corresponding 95% confidence intervals. The exact values by sample are presented in Table A2.

Modification	Sample	Class	Total Sites	Exon (%)	Intron (%)	Intergenic (%)
6mA	Young	Mono	212,250	4.9 ± 0.2	52.2 ± 0.8	42.8 ± 0.5
		Di	20,493	5.1 ± 1.3	54.0 ± 1.8	40.9 ± 2.6
		Tri	4356	5.5 ± 1.7	52.4 ± 11.0	42.0 ± 10.4
		Tetra	1359	5.1 ± 6.3	53.8 ± 1.5	41.1 ± 5.2
	Old	Mono	189,707	4.9 ± 0.3	51.7 ± 0.5	43.4 ± 0.8
		Di	18,148	5.2 ± 0.7	53.2 ± 2.3	41.6 ± 1.9
		Tri	3645	4.7 ± 0.6	52.1 ± 1.6	43.2 ± 1.8
		Tetra	1128	4.5 ± 3.8	49.6 ± 18.0	45.6 ± 19.6
5mC	Young	Mono	765,373	8.4 ± 0.2	52.0 ± 0.2	39.6 ± 0.3
		Di	98,032	8.9 ± 1.7	52.3 ± 1.3	38.8 ± 0.7
		Tri	21,806	9.0 ± 2.3	50.2 ± 7.7	40.8 ± 6.0
		Tetra	7058	9.0 ± 3.9	49.2 ± 5.8	41.7 ± 5.9
	Old	Mono	650,811	8.2 ± 0.8	51.9 ± 0.9	40.0 ± 0.8
		Di	86,985	8.5 ± 0.8	52.1 ± 3.7	39.3 ± 2.9
		Tri	19,198	8.6 ± 5.7	49.1 ± 0.8	42.3 ± 6.6
		Tetra	5828	8.4 ± 7.1	50.7 ± 8.8	41.0 ± 9.6
5hmC	Young	Mono	30,923	8.4 ± 0.5	56.9 ± 1.8	34.7 ± 1.7
		Di	4372	9.3 ± 4.0	59.3 ± 5.4	31.4 ± 2.5
		Tri	951	12.2 ± 10.7	56.3 ± 8.8	31.4 ± 10.8
		Tetra	319	9.3 ± 22.7	58.4 ± 26.1	32.3 ± 22.1
	Old	Mono	25,016	7.5 ± 1.7	56.4 ± 4.5	36.1 ± 3.2
		Di	3331	7.8 ± 4.8	59.5 ± 10.6	32.6 ± 6.0
		Tri	720	9.1 ± 2.0	60.6 ± 5.7	30.3 ± 3.8
		Tetra	206	8.9 ± 11.6	53.4 ± 16.4	37.7 ± 6.6

**Table 8:** Genomic distribution of cfDNA fragments across exonic, intronic, and intergenic regions in different nucleosomal fragment classes. Values are presented as mean percentages across biological replicates with corresponding 95% confidence intervals. The exact values by sample are presented in Table A3.

Sample	Class	Total Reads	Exon (%)	Intron (%)	Intergenic (%)
Young	Mono	586,697	6.8 ± 0.2	53.5 ± 0.4	42.8 ± 0.5
	Di	29,926	9.4 ± 0.9	55.3 ± 1.4	41.3 ± 2.2
	Tri	4091	11.5 ± 2.7	54.4 ± 7.6	43.0 ± 8.2
	Tetra	1009	12.7 ± 4.1	55.1 ± 2.6	43.3 ± 2.5
Old	Mono	495,784	6.6 ± 0.3	53.4 ± 1.2	43.0 ± 1.2
	Di	25,954	8.3 ± 2.2	55.0 ± 2.3	41.6 ± 2.4
	Tri	3463	10.9 ± 2.1	53.9 ± 1.5	43.3 ± 2.2
	Tetra	842	12.0 ± 3.9	55.3 ± 5.5	41.0 ± 3.5

**Table 9:** Sample metadata. 3 old and 3 young individuals.

Sample_ID	Gender	Feature	Age
young_fem_1160_batch1	female	young	18
young_fem_986_batch1	female	young	20
young_fem_1178_batch1	female	young	27
old_fem_1107_batch1	female	old	81
old_male_989_batch1	male	old	84
old_male_1158_batch1	male	old	85

**Table 10:** Control DNA samples based on Raji genomic DNA and their preparation scheme.

Sample ID	M.SssI Treatment	DNase I Pretreatment	AluI/HaeIII Treatment	cfDNA Extraction from Surrogate Plasma Buffer
dna_raji_HaeIII_AluI_batch4	−	−	+	–
dna_raji_dnase1_HaeIII_AluI_batch4	−	+	+	−
methylated_dna_raji_HaeIII_AluI_batch4	+	−	+	−
methylated_dna_raji_dnase1_HaeIII_AluI_batch4	+	+	+	−
dna_raji_HaeIII_AluI_batch6	−	+	+	+
dna_raji_methyl_HaeIII_AluI_batch6	+	+	+	+
dna_raji_HaeIII_AluI_C_batch6	−	+	+	−
dna_raji_methyl_HaeIII_AluI_C_batch6	+	+	+	−

**Figure A1:**
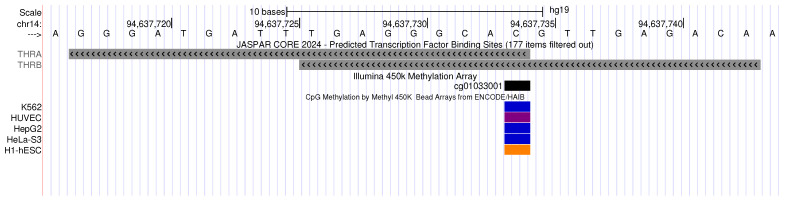
Genomic context of the age-associated CpG site cg01033001. UCSC Genome Browser view showing the position of cg01033001 relative to predicted transcription factor binding sites from JASPAR CORE 2024, including THRA and THRB motifs, and CpG methylation tracks from ENCODE/HAIB across multiple cell types (K562, HUVEC, HepG2, HeLa-S3, and H1-hESC). The locus overlaps a predicted transcription factor binding region, suggesting potential regulatory relevance.

**Figure A2:**
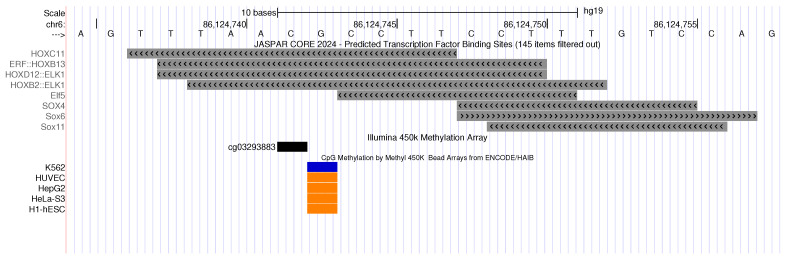
Genomic context of the age-associated CpG site cg03293883. UCSC Genome Browser view showing the position of cg03293883 relative to predicted transcription factor binding sites from JASPAR CORE 2024, including HOXC11, ERF::HOXB13, HOXD12::ELK1, HOXB2::ELK1, ELF5, and SOX-family motifs (SOX4, SOX6, and SOX11), as well as CpG methylation tracks from ENCODE/HAIB across multiple cell types (K562, HUVEC, HepG2, HeLa-S3, and H1-hESC). The locus overlaps a dense cluster of predicted transcription factor binding motifs, suggesting potential regulatory relevance.

**Figure A3:**
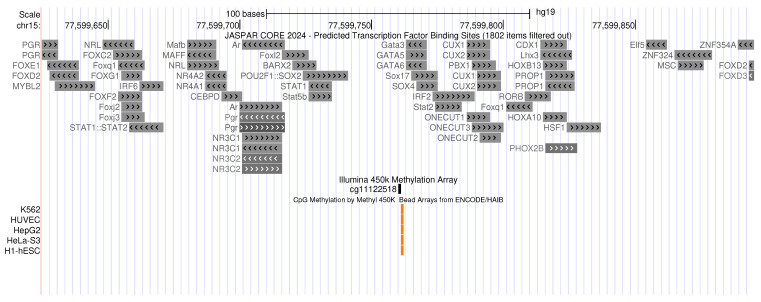
Genomic context of the age-associated CpG site cg11122518. UCSC Genome Browser view showing the position of cg11122518 relative to a broad cluster of predicted transcription factor binding sites from JASPAR CORE 2024, including FOX-, SOX-, GATA-, STAT-, CUX-, HOX-, and nuclear receptor-related motifs, as well as CpG methylation tracks from ENCODE/HAIB across multiple cell types (K562, HUVEC, HepG2, HeLa-S3, and H1-hESC). The locus is positioned within a dense predicted regulatory region enriched in overlapping transcription factor motifs, suggesting potential regulatory relevance of age-associated methylation changes at this site.

## Data Availability

The sequencing reads generated in this study have been submitted to the NCBI BioProject database https://www.ncbi.nlm.nih.gov/bioproject/ (accessed on 22 June 2026) under accession number PRJNA1429017.

## Abbreviations

The following abbreviations are used in this manuscript:

cfDNA	cell-free DNA
ctDNA	circulating tumor DNA
5mC	5-methylcytosine
5hmC	5-hydroxymethylcytosine
6mA	N6-methyladenine
CpG	cytosine-phosphate-guanine dinucleotide
LINE-1	long interspersed nuclear element 1
T2T	telomere-to-telomere
BAM	binary alignment/map format
BED	browser extensible data format
GFF3	general feature format version 3
FDR	false discovery rate
Q30	Phred quality score of 30

## Appendix A

**Table A1:** Inter-batch technical variability estimated from pairwise deviation between matched samples from independent experimental runs (batch4 vs. batch6). Absolute deviation was calculated as $\Delta =|x_{1}-x_{2}|$. Relative technical variability was calculated as $\frac{|x_{1}-x_{2}|}{(x_{1}+x_{2})/2}\times 100$.

Compared Samples	5mC Δ	5mC (%)	5hmC Δ	5hmC (%)	6mA Δ	6mA (%)
dna_raji_HaeIII_AluI_batch4 vs. dna_raji_HaeIII_AluI_batch6	0.134	3.82	0.01354	13.96	0.77040	158.0
methylated_dna_raji_HaeIII_AluI_batch4 vs. dna_raji_methyl_HaeIII_AluI_batch6	0.554	12.43	0.01640	15.54	0.75234	160.4

**Table A2:** Genomic distribution of 6mA, 5mC, and 5hmC modification sites across exonic, intronic, and intergenic regions for individual biological samples and nucleosomal fragment classes. Percentages are calculated relative to the total number of modification sites in the corresponding sample, modification type, and nucleosomal fragment class.

Sample	Modification	Class	Total Sites	Exon	Intron	Intergenic
young_fem_1178_batch1	6mA	mono	71,842	3633 (5.1%)	37,264 (51.9%)	30,943 (43.1%)
		di	8107	422 (5.2%)	4442 (54.8%)	3243 (40.0%)
		tri	1751	84 (4.8%)	980 (56.0%)	687 (39.2%)
		tetra	512	41 (8.0%)	273 (53.3%)	198 (38.7%)
	5mC	mono	249,616	20,695 (8.3%)	129,865 (52.0%)	99,056 (39.7%)
		di	37,550	3218 (8.6%)	19,780 (52.7%)	14,552 (38.8%)
		tri	8209	657 (8.0%)	4393 (53.5%)	3158 (38.5%)
		tetra	2587	193 (7.5%)	1325 (51.2%)	1069 (41.3%)
	5hmC	mono	11,125	911 (8.2%)	6410 (57.6%)	3803 (34.2%)
		di	1873	197 (10.5%)	1097 (58.6%)	579 (30.9%)
		tri	363	31 (8.5%)	218 (60.1%)	113 (31.1%)
		tetra	128	6 (4.7%)	90 (70.3%)	32 (25.0%)
young_fem_1160_batch1	6mA	mono	79,095	3863 (4.9%)	41,394 (52.3%)	33,838 (42.8%)
		di	7173	395 (5.5%)	3853 (53.7%)	2922 (40.7%)
		tri	1295	80 (6.2%)	697 (53.8%)	518 (40.0%)
		tetra	401	16 (4.0%)	215 (53.6%)	170 (42.4%)
	5mC	mono	291,327	24,579 (8.4%)	151,326 (51.9%)	115,422 (39.6%)
		di	35,323	3425 (9.7%)	18,263 (51.7%)	13,635 (38.6%)
		tri	7353	716 (9.7%)	3654 (49.7%)	2983 (40.6%)
		tetra	2119	224 (10.6%)	1056 (49.8%)	839 (39.6%)
	5hmC	mono	12,068	1034 (8.6%)	6877 (57.0%)	4157 (34.4%)
		di	1669	164 (9.8%)	962 (57.6%)	543 (32.5%)
		tri	390	66 (16.9%)	218 (55.9%)	106 (27.2%)
		tetra	101	20 (19.8%)	51 (50.5%)	30 (29.7%)
young_fem_986_batch1	6mA	mono	61,313	2995 (4.9%)	32,161 (52.5%)	26,155 (42.7%)
		di	5213	235 (4.5%)	2783 (53.4%)	2194 (42.1%)
		tri	1310	74 (5.6%)	622 (47.5%)	614 (46.9%)
		tetra	446	15 (3.4%)	243 (54.5%)	188 (42.2%)
	5mC	mono	224,430	18,836 (8.4%)	117,008 (52.1%)	88,586 (39.5%)
		di	25,159	2117 (8.4%)	13,199 (52.5%)	9843 (39.1%)
		tri	6244	584 (9.4%)	2956 (47.3%)	2704 (43.3%)
		tetra	2352	213 (9.1%)	1097 (46.6%)	1042 (44.3%)
	5hmC	mono	7730	645 (8.3%)	4345 (56.2%)	2740 (35.4%)
		di	830	62 (7.5%)	513 (61.8%)	255 (30.7%)
		tri	198	22 (11.1%)	105 (53.0%)	71 (35.9%)
		tetra	90	3 (3.3%)	49 (54.4%)	38 (42.2%)
old_male_989_batch1	6mA	mono	67,832	3282 (4.8%)	34,954 (51.5%)	29,596 (43.6%)
		di	7055	362 (5.1%)	3717 (52.7%)	2976 (42.2%)
		tri	1706	80 (4.7%)	875 (51.3%)	751 (44.0%)
		tetra	579	30 (5.2%)	332 (57.3%)	217 (37.5%)
	5mC	mono	231,588	19,338 (8.4%)	119,152 (51.4%)	93,097 (40.2%)
		di	32,458	2742 (8.4%)	16,924 (52.1%)	12,792 (39.4%)
		tri	8473	767 (9.1%)	4163 (49.1%)	3543 (41.8%)
		tetra	2819	308 (10.9%)	1334 (47.3%)	1177 (41.8%)
	5hmC	mono	9889	782 (7.9%)	5583 (56.5%)	3524 (35.6%)
		di	1382	119 (8.6%)	788 (57.0%)	475 (34.4%)
		tri	318	30 (9.4%)	190 (59.7%)	98 (30.8%)
		tetra	104	6 (5.8%)	62 (59.6%)	36 (34.6%)
old_fem_1107_batch1	6mA	mono	113,033	5440 (4.8%)	58,317 (51.6%)	49,273 (43.6%)
		di	10,065	555 (5.5%)	5297 (52.6%)	4213 (41.9%)
		tri	1649	82 (5.0%)	865 (52.5%)	702 (42.6%)
		tetra	442	25 (5.7%)	214 (48.4%)	203 (45.9%)
	5mC	mono	391,702	32,721 (8.4%)	203,859 (52.0%)	155,122 (39.6%)
		di	50,194	4458 (8.9%)	25,430 (50.7%)	20,306 (40.5%)
		tri	9308	989 (10.6%)	4600 (49.4%)	3719 (40.0%)
		tetra	2403	214 (8.9%)	1306 (54.3%)	883 (36.7%)
	5hmC	mono	13,829	1085 (7.8%)	7556 (54.6%)	5188 (37.5%)
		di	1735	160 (9.2%)	991 (57.1%)	584 (33.7%)
		tri	353	34 (9.6%)	208 (58.9%)	111 (31.4%)
		tetra	74	5 (6.8%)	40 (54.1%)	29 (39.2%)
old_male_1158_batch1	6mA	mono	8842	442 (5.0%)	4591 (51.9%)	3809 (43.1%)
		di	1028	51 (5.0%)	558 (54.3%)	419 (40.8%)
		tri	290	13 (4.5%)	152 (52.4%)	125 (43.1%)
		tetra	107	3 (2.8%)	46 (43.0%)	57 (53.3%)
	5mC	mono	27,521	2152 (7.8%)	14,344 (52.1%)	11,025 (40.1%)
		di	4333	357 (8.2%)	2324 (53.6%)	1652 (38.1%)
		tri	1417	86 (6.1%)	691 (48.8%)	640 (45.2%)
		tetra	606	32 (5.3%)	305 (50.3%)	269 (44.4%)
	5hmC	mono	1298	87 (6.7%)	756 (58.2%)	455 (35.1%)
		di	214	12 (5.6%)	138 (64.5%)	64 (29.9%)
		tri	49	4 (8.2%)	31 (63.3%)	14 (28.6%)
		tetra	28	4 (14.3%)	13 (46.4%)	11 (39.3%)

**Table A3:** Genomic distribution of cfDNA fragments across exonic, intronic, and intergenic regions for individual biological samples and nucleosomal fragment classes.

Sample	Class	Total Reads	Exon Reads	Intron Reads	Intergenic Reads
young_fem_1178_batch1	mono	195,387	13,372 (6.8%)	104,780 (53.6%)	83,256 (42.6%)
	di	11,765	1131 (9.6%)	6582 (55.9%)	4739 (40.3%)
	tri	1617	179 (11.1%)	922 (57.0%)	644 (39.8%)
	tetra	381	53 (13.9%)	212 (55.6%)	161 (42.3%)
young_fem_1160_batch1	mono	221,801	14,993 (6.8%)	118,223 (53.3%)	95,307 (43.0%)
	di	10,993	1027 (9.3%)	6053 (55.1%)	4585 (41.7%)
	tri	1437	182 (12.7%)	748 (52.1%)	620 (43.1%)
	tetra	379	43 (11.3%)	215 (56.7%)	159 (42.0%)
young_fem_986_batch1	mono	169,509	11,393 (6.7%)	90,722 (53.5%)	72,426 (42.7%)
	di	7168	668 (9.3%)	3929 (54.8%)	3004 (41.9%)
	tri	1037	109 (10.5%)	567 (54.7%)	482 (46.5%)
	tetra	249	33 (13.3%)	129 (51.8%)	116 (46.6%)
old_male_989_batch1	mono	187,343	12,598 (6.7%)	99,931 (53.3%)	80,606 (43.0%)
	di	10,397	905 (8.7%)	5673 (54.6%)	4381 (42.1%)
	tri	1385	149 (10.8%)	752 (54.3%)	597 (43.1%)
	tetra	359	39 (10.9%)	192 (53.5%)	146 (40.7%)
old_fem_1107_batch1	mono	278,464	18,679 (6.7%)	148,641 (53.4%)	120,745 (43.4%)
	di	14,682	1273 (8.7%)	8070 (55.0%)	6081 (41.4%)
	tri	1785	206 (11.5%)	978 (54.8%)	747 (41.8%)
	tetra	395	53 (13.4%)	232 (58.7%)	154 (39.0%)
old_male_1158_batch1	mono	29,977	1860 (6.2%)	14,884 (49.6%)	12,658 (42.2%)
	di	875	85 (9.7%)	398 (45.5%)	353 (40.3%)
	tri	293	29 (9.9%)	137 (46.8%)	142 (48.5%)
	tetra	88	14 (15.9%)	47 (53.4%)	44 (50.0%)
